# ACR11 modulates levels of reactive oxygen species and salicylic acid-associated defense response in Arabidopsis

**DOI:** 10.1038/s41598-018-30304-0

**Published:** 2018-08-07

**Authors:** Shashi Kant Singh, Tzu-Ying Sung, Tsui-Yun Chung, Shao-Yu Lin, Sang-Chu Lin, Jo-Chien Liao, Wei-Yu Hsieh, Ming-Hsiun Hsieh

**Affiliations:** Institute of Plant and Microbial Biology, Academia Sinica, Taipei, 11529 Taiwan

## Abstract

The ACT domain (aspartate kinase, chorismate mutase and TyrA), an allosteric effector binding domain, is commonly found in amino acid metabolic enzymes. In addition to ACT domain-containing enzymes, plants have a novel family of ACT domain repeat (ACR) proteins, which do not contain any recognizable catalytic domain. Arabidopsis has 12 ACR proteins, whose functions are largely unknown. To study the functions of Arabidopsis ACR11, we have characterized two independent T-DNA insertion mutants, *acr11-2* and *acr11-3*. RNA gel-blot analysis revealed that the expression of wild-type *ACR11* transcripts was not detectable in the *acr11* mutants. Interestingly, a lesion-mimic phenotype occurs in some rosette leaves of the *acr11* mutants. In addition, high levels of reactive oxygen species (ROS), salicylic acid (SA), and callose accumulate in the mutant leaves when grown under normal conditions. The expression of several SA marker genes and the key SA biosynthetic gene *ISOCHORISMATE SYNTHASE1* is up-regulated in the *acr11* mutants. Furthermore, the *acr11* mutants are more resistant to the infection of bacterial pathogen *Pseudomonas syringae* pathovar *tomato* DC3000. These results suggest that ACR11 may be directly or indirectly involved in the regulation of ROS and SA accumulation, which in turn modulates SA-associated defense responses and disease resistance in Arabidopsis.

## Introduction

Amino acids are essential organic compounds for all life forms. The synthesis of these important molecules is tightly regulated. It is well established that many key enzymes involved in amino acid biosynthesis are subject to feedback inhibition. These feedback-regulated enzymes are usually composed of catalytic domains and allosteric domains, which are responsible for catalyzing the reaction and allosteric regulation of the enzyme activity, respectively^1–4^. Interestingly, despite being feedback-regulated by different amino acids, the allosteric domain of these enzymes shares some common features in the primary sequence and tertiary structure, which has been named the ACT domain after bacterial aspartate kinase (AK), chorismate mutase (CM) and TyrA (prephenate dehydrogenase, PDH)^5,6^. AK catalyzes the first reaction of the biosynthesis of aspartate family amino acids, including lysine, methionine, and threonine. The activity of AK is feedback-regulated by lysine and threonine via the conserved ACT domain^7^. CM and PDH are involved in the biosynthesis of aromatic amino acids, which are feedback-regulated by phenylalanine and tyrosine through the regulatory ACT domain^1,8^.

Feedback regulation of amino acid biosynthetic enzymes has been extensively studied in bacteria. Interestingly, these enzymes are highly conserved from bacteria to plants. Most plant homologs have similar domain composition, e.g. a specific enzyme catalytic domain, followed by a general allosteric regulatory ACT domain^2,3,7^. It is likely that the ACT domain fused to the amino acid metabolic enzymes also serves as an allosteric ligand-binding domain in plants. Indeed, the Arabidopsis AK is feedback-inhibited by lysine and *S*-adenosylmethionine via the ACT domain^9^. The three committed enzymes in branched-chain amino acid BCAA biosynthesis, e.g. threonine deaminase, acetohydroxy acid synthase, and isopropylmalate synthase are feedback-regulated by branched-chain amino acids mainly via the ACT domains of these enzymes in Arabidopsis^10^. Furthermore, the Arabidopsis ACT domain-containing enzyme phosphoglycerate dehydrogenase is feedback regulated by its end product serine^11^. Thus, the ACT domain is a widespread allosteric regulatory domain that is highly conserved from bacteria to plants.

We previously identified a novel ACT domain repeat (ACR) protein family in Arabidopsis^2,12^. The ACR proteins contain only ACT domain repeats but not any recognizable catalytic domain. The functions of these plant ACR proteins are largely unknown. Arabidopsis has 12 ACR proteins, which are further divided into 3 different groups according to their ACT domain composition and sequence homology^12^. Group III ACR proteins, e.g. ACR11 and ACR12, are distinct in that they contain a non-conserved N-terminal transit peptide followed by two conserved ACT domains. Indeed, the Arabidopsis ACR11 and ACR12 proteins have been demonstrated to localize in the chloroplast^12^. The *ACR11* gene is specifically expressed in green tissues, and is coordinately regulated with *GLN2* encoding a chloroplastic glutamine synthetase 2 (GS2) in Arabidopsis^12^. Recently, the Arabidopsis ACR11 protein was shown to activate the activity of GS2 and levels of glutamine were significantly reduced in the *acr11* mutant^13^. In addition, the Arabidopsis ACR11 protein was shown to interact with ferredoxin-dependent glutamine oxoglutarate aminotransferase 1 (Fd-GOGAT1) and the activity of Fd-GOGAT was reduced in the *acr11* mutants^14^. It has been proposed that ACR11 may stabilize Fd-GOGAT and possibly modulates its activity^14^. Nevertheless, the molecular mechanisms of ACR11 have yet to be elucidated.

Here, we have characterized two independent *acr11* T-DNA insertion mutants in Arabidopsis. Interestingly, spontaneous cell death occurs in the rosette leaves of the *acr11* mutants. The lesion-mimic phenotype accompanied by increased levels of reactive oxygen species (ROS) and salicylic acid (SA)-associated defense responses make the *acr11* mutants more resistant to the bacterial pathogen *Pseudomonas syringae* pathvar *tomato* DC3000 (*Pst*). The homeostasis of glutamine has been proposed to modulate SA-associated redox status and defense responses in Arabidopsis^15^. The functions of Arabidopsis ACR11 in the interconnections of GS/Fd-GOGAT cycle, glutamine homeostasis, redox balance, ROS accumulation, and SA-associated defense responses are discussed herein.

## Results

### Isolation and characterization of Arabidopsis *acr11* mutants

**Figure 1:**
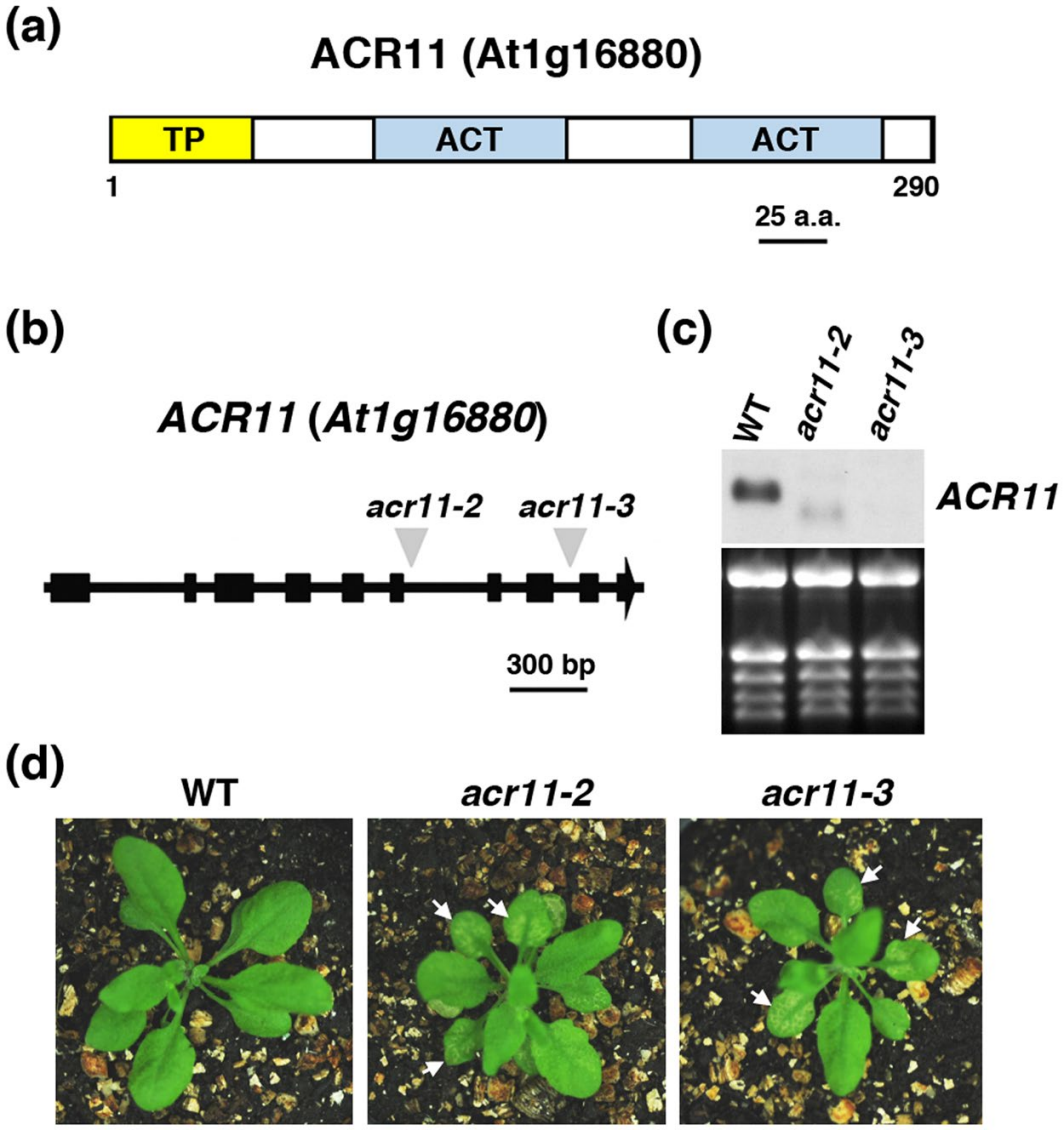
Molecular and phenotypic analyses of Arabidopsis *acr11* mutants. (**a**) Schematic diagram of the Arabidopsis ACR11 protein. TP, transit peptide. (**b**) Schematic diagram of the *ACR11* gene and the locations of T-DNA insertion in the *acr11-2* and *acr11-3* mutants. (**c**) RNA gel-blot analysis. Total RNA extracted from 2-week-old wild type (WT), *acr11-2* and *acr11-3* seedlings was used to detect the expression of *ACR11*. The full-length blot is shown in Supplementary Fig. S5. (**d**) Four-week-old Arabidopsis WT, *acr11-2* and *acr11-3* mutant plants grown in soil under normal conditions. Lesions appear in the rosette leaves of the *acr11* mutants are indicated by white arrows.

The Arabidopsis ACR11 protein is predicted to contain a transit peptide with the cleavage site located at the 52^nd^ residue (www.cbs.dtu.dk/services/TargetP/) followed by two ACT domains (Fig. 1a). To further characterize the functions of ACR11, we obtained two independent T-DNA insertion lines, SAIL_14H_10 and SALK_025722, from the Arabidopsis Biological Resource Center (ABRC). The *acr11* homozygous mutant plants were isolated from these T-DNA insertion lines by PCR and confirmed by genomic Southern blot analysis (Supplementary Fig. S1). The T-DNA mutant of SAIL_14H_10 was previously named *acr11-2*^14^. We have adopted the nomenclature and named the new allele derived from the SALK_025722 T-DNA line *acr11-3*. The positions of T-DNA insertion of *acr11-2* and *acr11-3* are shown in Fig. 1b. We used RNA gel-blot analysis to examine the expression of *ACR11* in wild-type, *acr11-2* and *acr11-3* seedlings. Although the wild-type *ACR11* transcript was not detectable in *acr11-2*, two faint bands, one higher and another lower than *ACR11*, were detected in the mutant (Fig. 1c). The identities of these two bands are unknown. By contrast, transcripts of *ACR11* were not detectable in the *acr11-3* mutant (Fig. 1c).

**Figure 2:**
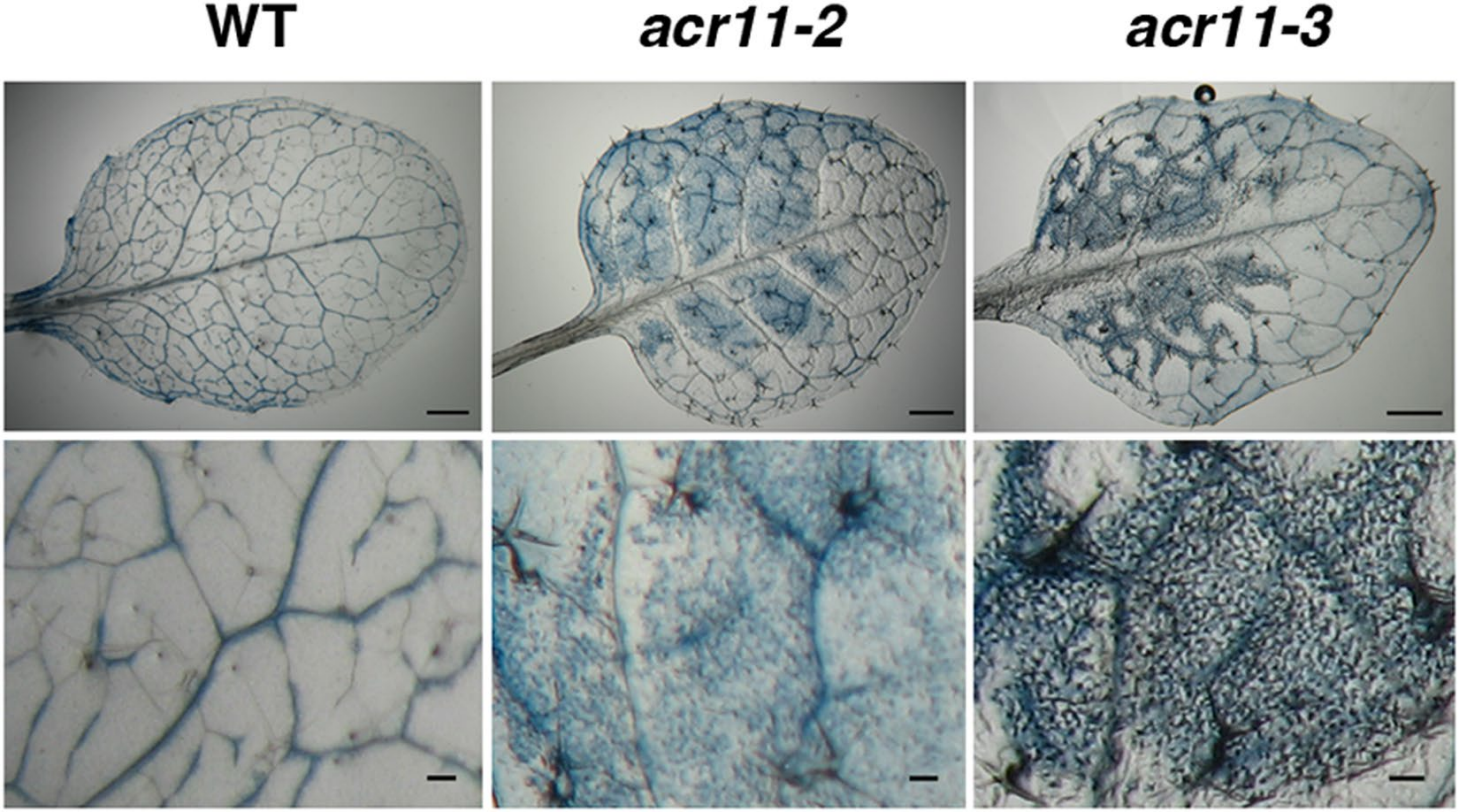
Trypan blue staining for the detection of cell death in the rosette leaves of 5-week-old Arabidopsis wild type (WT) and *acr11* mutants. Scale bars are 1 mm (top) and 0.1 mm (bottom).

The phenotypes of *acr11-2* and *acr11-3* are very similar. The mutant plants are smaller than wild type (Supplementary Fig. S2), and some lesions appear in the rosette leaves of *acr11-2* and *acr11-3* mutants when grown in soil under a 16-h light/8-h dark cycle (Fig. 1d). The lesion usually starts to develop in the rosette leaves of 3- to 4-week-old *acr11* mutant plants (Fig. 1d). We used trypan blue to stain dead cells in the lesion-containing rosette leaves from *acr11-2* and *acr11-3*. Compared with the wild type, the *acr11* mutants possess more dead cells in the rosette leaves (Fig. 2). Thus, the lesions occur in some of the rosette leaves can be attributed to spontaneous cell death in the *acr11* mutants.

### Accumulation of ROS and callose in the *acr11* mutants

**Figure 3:**
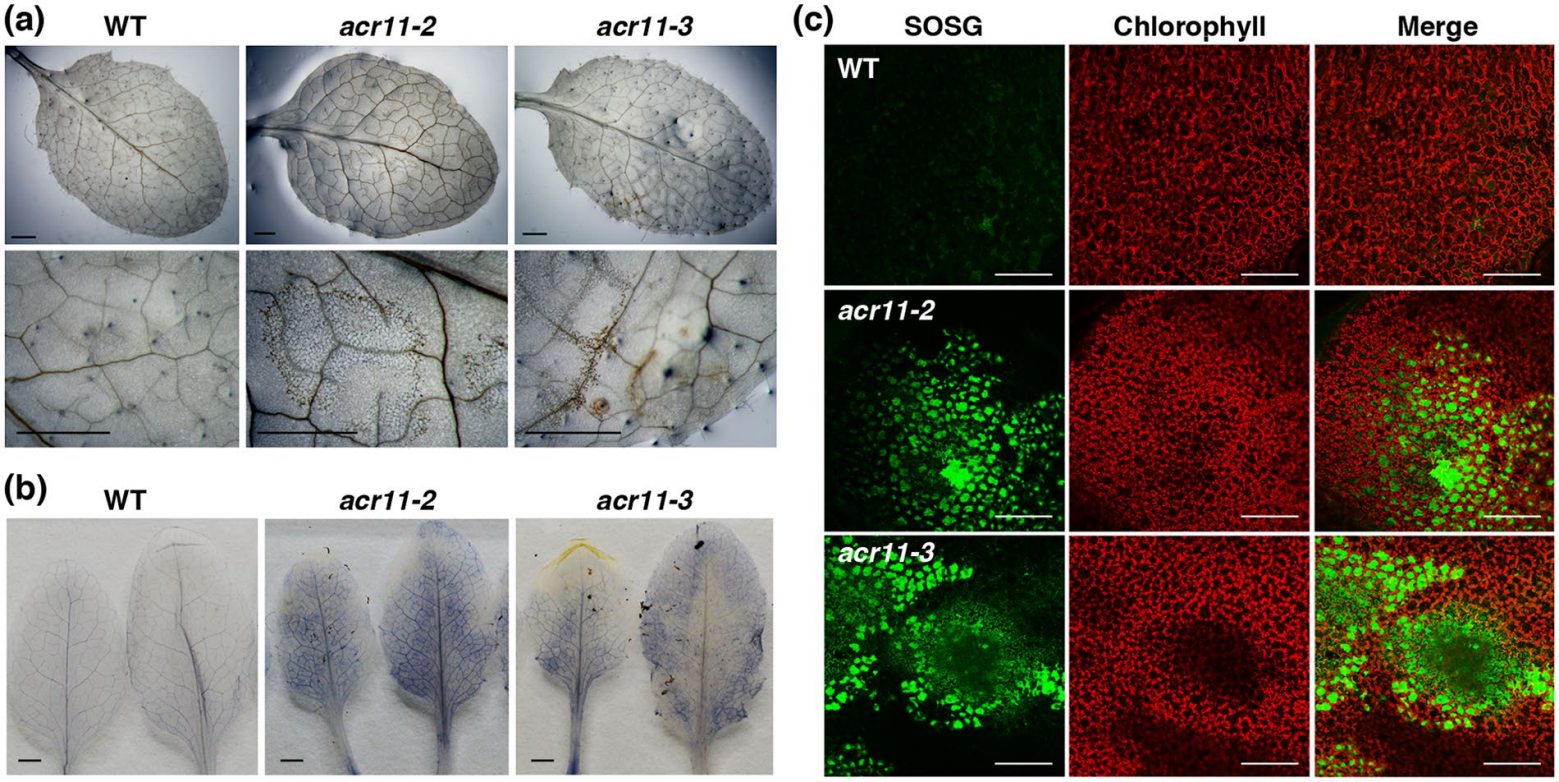
Comparison of reactive oxygen species levels in the rosette leaves of 5-week-old Arabidopsis wild-type (WT) and *acr11* mutant plants. (**a**) Staining of hydrogen peroxide by 3,3′-diaminobenzidine. (**b**) Staining of superoxide radical by nitroblue tetrazolium. (**c**) Staining of singlet oxygen species by Singlet Oxygen Sensor Green (SOSG) fluorescent dye. Scale bars are 1 mm in (**a**) and (**b**), 200 μm in (**c**).

**Figure 4:**
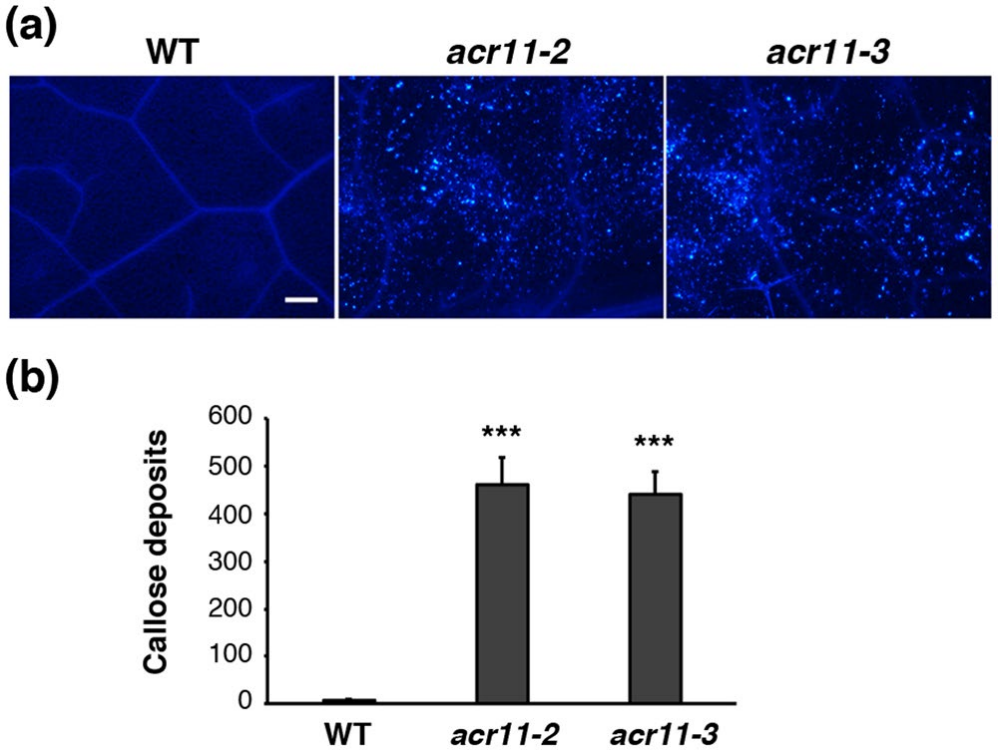
Callose deposition in the rosette leaves of 5-week-old Arabidopsis wild type (WT) and *acr11* mutants. (**a**) Staining of callose deposits by aniline blue. Scale bar is 100 μm. (**b**) Quantification of callose deposits in WT and *acr11* mutant leaves. Values shown are means ± SD per 0.97 mm^2^ from leaves of 5 independent plants. ***P < 0.001 represents the result of Student’s *t* test.

It is known that accumulation of ROS commonly proceeds spontaneous cell death in plants^16^. We thus examined the accumulation of ROS, including H_2_O_2_, superoxide, and singlet oxygen species, in the rosette leaves of 5-week-old wild-type and *acr11* mutant plants by diaminobenzidine tetrahydrochloride (DAB), nitroblue tetrazolium (NBT), and Singlet Oxygen Sensor Green (SOSG) staining, respectively. Levels of H_2_O_2_, superoxide, and singlet oxygen are significantly higher in the mutant leaves as compared with those of the wild-type (Fig. 3a–c). These results suggest that the *acr11* mutants have accumulated excessive amounts of ROS when grown under normal conditions. In addition, we used aniline blue to stain callose in the rosette leaves from 5-week-old wild-type and *acr11* mutant plants (Fig. 4a). The mutant leaves have accumulated significant amount of callose as compared with that of the wild type (Fig. 4a,b).

### The expression of SA marker genes is induced in the *acr11* mutants

**Figure 5:**
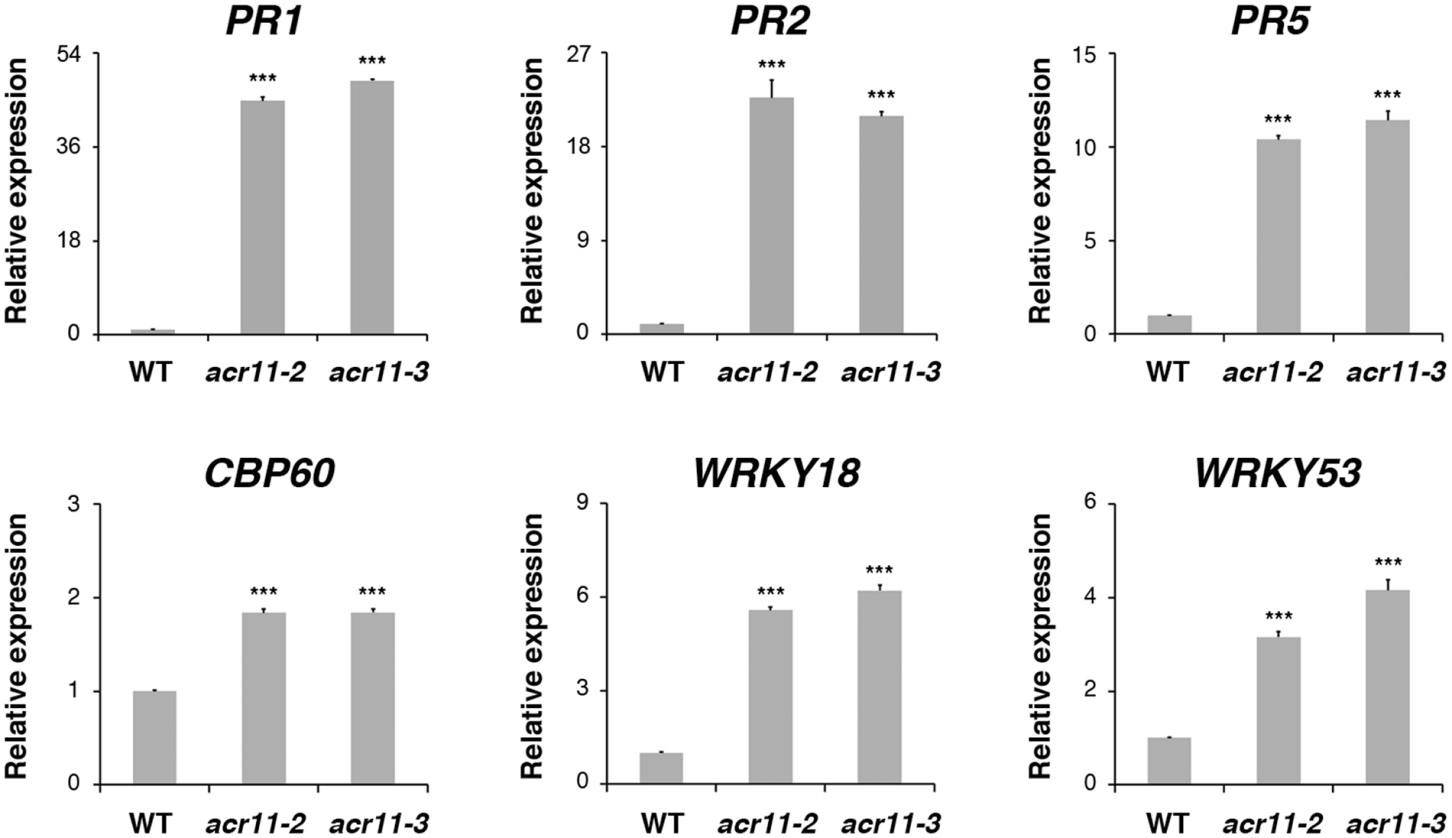
Quantitative RT-PCR analysis of salicylic acid-responsive genes. Total RNA extracted from rosette leaves of 5-week-old Arabidopsis wild-type (WT), *acr11-2*, and *acr11-3* mutant plants was used for qRT-PCR analysis to detect the expression of *PR1*, *PR2*, *PR5*, *CBP60G*, *WRKY18* and *WRKY53*. Relative expression indicates the fold-change of each gene as compared to that of WT. Results shown are means ± SD from three independent experiments. ***P < 0.001 represents the result of Student’s *t* test.

The lesion-mimic phenotype and accumulation of ROS in the rosette leaves suggest that the SA-related signaling pathways may be activated in the *acr11* mutants. To examine if the SA-associated responses are enhanced in the *acr11* mutants, we used quantitative (q) RT-PCR analysis to measure the expression of SA marker genes in the rosette leaves of 5-week-old wild type and *acr11* mutants. The selected SA maker genes include *PATHOGENESIS-RELATED 1* (*PR1*), *PR2*, *PR5*, *CALMODULIN BINDING PROTEIN 60 G* (*CBP60G*) and two WRKY transcription factor genes, *WRKY18* and *WRKY53*. Steady-state mRNA levels of *PR1*, *PR2*, *PR5*, *CBP60*, *WRKY18*, and *WRKY53* were significantly higher in the *acr11-2* and *acr11-3* mutants as compared with those of the wild type (Fig. 5). In addition, RNA gel-blot analysis of *PR1* and *PR2* in Arabidopsis wild type and *acr11-3* was shown in Supplementary Fig. S3. These results indicate that the SA-associated responses are constitutively activated in the *acr11* mutant rosette leaves.

### Accumulation of SA in the *acr11* mutants

**Figure 6:**
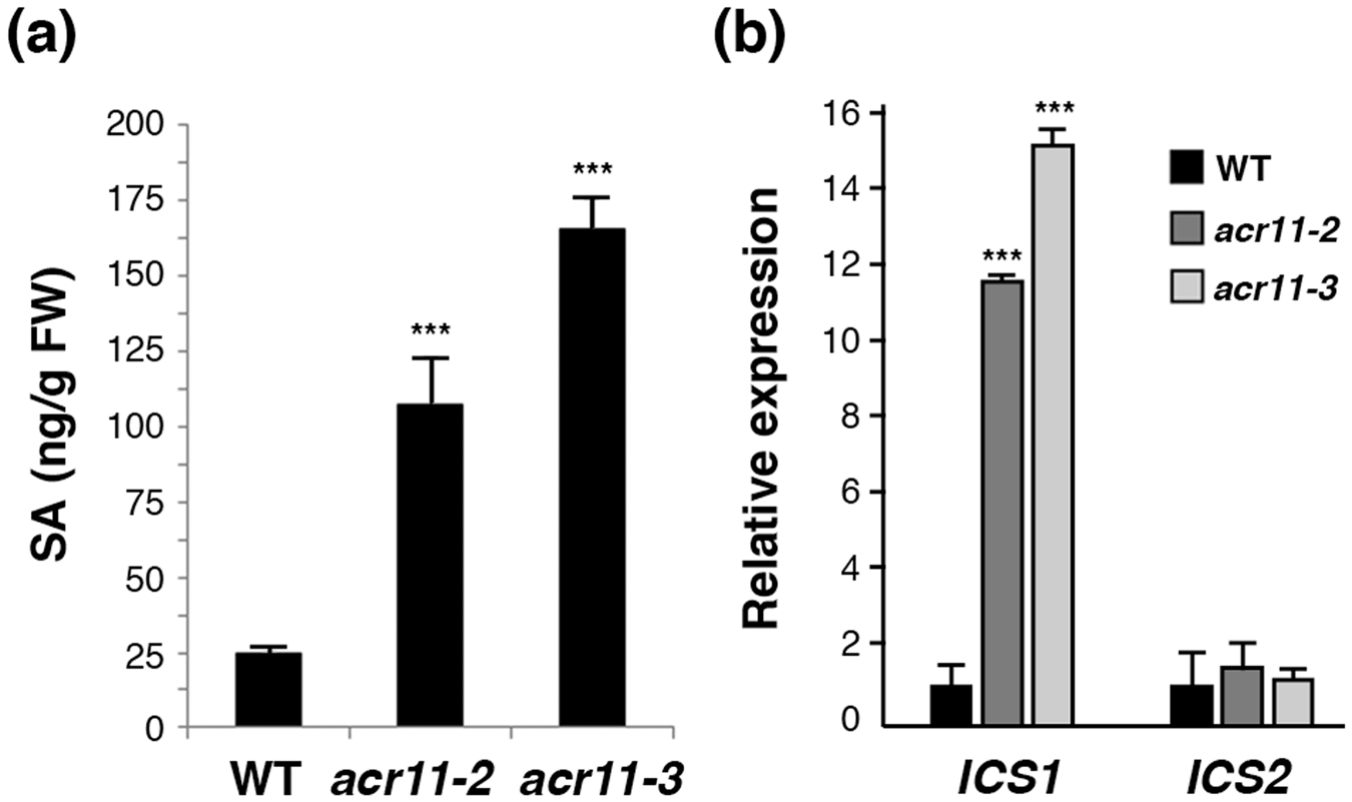
Enhanced salicylic acid (SA) accumulation is in the *acr11* mutants. (**a**) Levels of free SA in the rosette leaves of 5-week-old Arabidopsis wild type (WT) and *acr11* mutants. (**b**) Quantitative RT-PCR analysis of SA biosynthetic genes in the rosette leaves of 5-week-old Arabidopsis WT and *acr11* mutants. The expression levels of *ICS1* and *ICS2* in WT were set at 1. Fold change indicates the relative expression of *ICS1* and *ICS2* as compared to that of WT. *ICS*, *ISOCHORISMATE SYNTHASE*. Results shown are means ± SD from three independent experiments. ***P < 0.001 represents the result of Student’s *t* test.

Perturbation of endogenous SA levels will directly affect the SA-associated responses in plants. To test if the constitutively activated SA responses in the *acr11* mutants is caused by changes of SA levels, we measured the amount of SA in the rosette leaves of 5-week-old wild-type, *acr11-2* and *acr11-3* mutant plants. The results indicate that the SA levels in the mutant rosette leaves are significantly higher than those of the wild type (Fig. 6a). It is known that isochorismate synthase (ICS) is the major enzyme involved in SA biosynthesis in Arabidopsis^17^. There are two *ICS* genes, *ICS1* and *ICS2*, in Arabidopsis. We used qRT-PCR analysis to examine the expression of *ICS1* and *ICS2* in the rosette leaves of wild-type and *acr11* mutant plants. The expression levels of *ICS1*, but not *ICS2*, are much higher in the *acr11-2* and *acr11-3* mutants as compared to the wild type (Fig. 6b).

### Enhanced disease resistance in the *acr11* mutants

**Figure 7:**
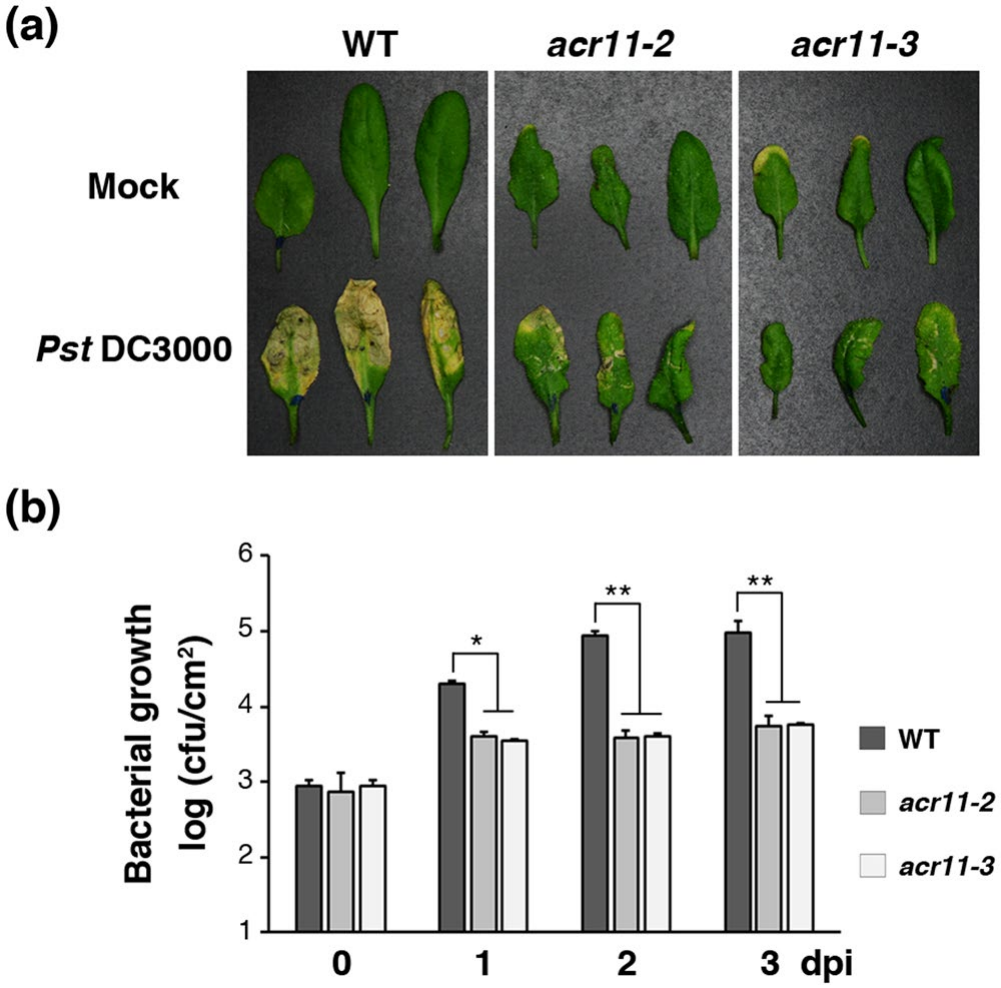
Enhanced disease resistance in 5-week-old Arabidopsis *acr11* mutants. (**a**) Symptoms of wild-type (WT), *acr11-2* and *acr11-3* rosette leaves 3 days after syringe infiltration with *Pseudomonas syringae* pv. *tomato* DC3000 (*Pst*). (**b**) Growth of *Pst* in Arabidopsis WT, *acr11-2* and *acr11-3* mutants. Bacterial titers were evaluated at 0 to 3 days post inoculation (dpi). Results are means ± SD from three independent experiments. Asterisks indicate significant differences (*P < 0.05; **P < 0.01; Student’s *t* test) compared to the WT.

The phenotypes of lesion-mimic, enhanced SA-dependent responses, and accumulation of callose, ROS and SA suggest that the *acr11* mutants may be more resistant to pathogen infection. To test this possibility, we used the virulence strain *Pseudomonas syringae* pathovar *tomato* DC3000 (*Pst*) to infect the rosette leaves of 5-week-old wild-type and *acr11* mutant plants with syringe infiltration. The disease symptoms developed in the mutant leaves were significantly weaker than those of the wild type 3 days after inoculation (Fig. 7a). This phenotype was associated with bacterial growth in leaves infiltrated with *Pst*. The number of bacteria growing in the mutant leaves was significantly smaller than that of the wild type 1 to 3 days post inoculation (Fig. 7b).

In addition to syringe infiltration on individual rosette leaves, we also inoculated whole plants with *Pst* by dipping. Compared with the wild type, the *acr11* mutant plants showed less severe disease symptoms 3 days after dip inoculation (Supplementary Fig. S4a). The rosette leaves of the *acr11* mutants had less bacterial growth as compared with that of the wild type 1 to 3 days after dip inoculation (Supplementary Fig. S4b). These results suggest that the *acr11* mutants are more resistant to *Pst* infection.

## Discussion

The prediction that plant ACR11 homologs contain two ACT domains is reminiscent of the recent discovery of human arginine sensor CASTOR1^18,19^. Unlike CASTOR1, the functions of plant ACR proteins are largely unknown. Nonetheless, these proteins share a common feature that they all contain multiple ACT domains, but not any recognizable catalytic domain. The human CASTOR1 and its homolog CASTOR2 are predicted to contain 2 ACT domains^18^. However, crystal structure analysis of the arginine-bound CASTOR1 reveals that it is in fact composed of 4 tandem ACT domains^19–21^. Therefore, the real composition of ACT domains in the ACR11 protein requires further studies on the crystal structure.

It is interesting that the Arabidopsis *acr11* mutants have a lesion-mimic phenotype. Usually, the lesion first appears in the rosette leaves of 3- to 4-week-old *acr11* mutant plants when grown in soil under a normal condition. Many plant lesion-mimic mutants are associated with disease resistance^22,23^. The spontaneous cell death phenotype and the induction of defense response are coordinated in the *acr11* mutants. Furthermore, we have shown that the Arabidopsis *acr11* mutants are more resistant to the bacterial pathogen *Pst* (Fig. 7 and Supplementary Fig. S4). These results suggest that the cell death pathway activated in the *acr11* mutants is interconnected with the defense pathway against *Pst*.

The ACR11 protein is localized to the chloroplast^12^, which is also the site for SA biosynthesis and one of the major sites for ROS production inside the plant cell. The increased disease resistance of the *acr11* mutants can be attributed to the accumulation of ROS and SA, which is well-documented to enhance plant immunity^17,24,25^. In the rosette leaves of Arabidopsis *acr11* mutants, levels of ROS, SA and callose are significantly increased as compared to those of the wild type (Figs 3, 4 and 6). Furthermore, the expression of SA biosynthetic and responsive genes is dramatically induced in the *acr11* mutant (Figs 5, 6). These results are consistent with the phenotype that the *acr11* mutant is more resistant to pathogen infection. Loss-of-function in ACR11 will increase ROS levels and activate defense responses in the plant. However, if the amounts of ROS accumulated inside the cell are over the threshold, cell death will occur in the *acr11* mutants. The molecular mechanisms of ACR11 in the chloroplast-triggered spontaneous cell death remain to be elucidated.

Alternatively, the increased disease resistance of the *acr11* mutants can be attributed to shortage of nutrients, e.g. glutamine and its derivatives, for bacterial growth in the host plant. Pathogens have to obtain their nitrogen nutrients from the host plant. Thus, the nitrogen status of the host plant is tightly associated with pathogenesis^26,27^. Interestingly, the pathogenic *Pst* has been shown to selectively catabolize abundant amino acids, such as glutamine, glutamate, and aspartate from the host plant^28^. The Arabidopsis *acr11* mutants have reduced levels of glutamine^13^. It is possible that decreased levels of glutamine may cause shortage of nutrients for bacterial growth, and thus confers pathogen resistance in the *acr11* mutants.

**Figure 8:**
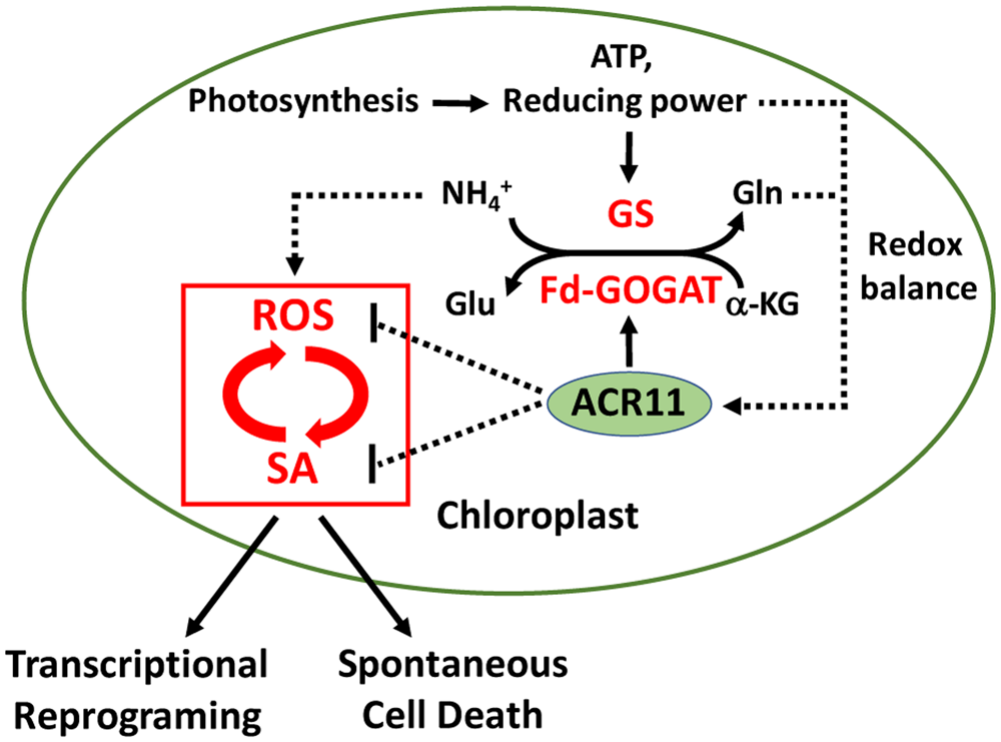
A hypothetical working model of ACR11 function in modulating reactive oxygen species (ROS) production and salicylic acid (SA)-associated defense responses. ACR11 may integrate the information of glutamine homeostasis and redox balance to modulate the activities of glutamine synthetase (GS)/ferredoxin-dependent glutamine oxoglutarate aminotransferase (Fd-GOGAT) and ROS production in the chloroplast. Overproduction of ROS activates SA biosynthesis and enhances SA-associated defense responses. In addition, ACR11 may directly affect SA biosynthesis, which in turn affects ROS production and SA-associated defense responses.

In addition to its role in nutrition and metabolism, glutamine can also function as a signaling molecule in plants^29,30^. It has been shown that glutamine homeostasis can modulate SA-associated redox status and defense responses in Arabidopsis^15^. It is conceivable that ACR11 may be involved in the maintenance of Gln homeostasis in Arabidopsis. We propose a hypothetical working model that Gln-deficiency derived from a compromised GS/Fd-GOGAT cycle may affect redox balance and induce ROS production in the *acr11* mutant (Fig. 8). This hypothesis may be partly supported by the following observations: (1) the *ACR11* and *GLN2* genes are coordinately expressed^12^; (2) ACR11 can activate GS2 and levels of glutamine are decreased in the *acr11* mutant^13^; (3) ACR11 interacts with Fd-GOGAT1 and the activity of Fd-GOGAT is reduced in the *acr11* mutant^14^. Arabidopsis Fd-GOGAT1 plays a major role in the assimilation of ammonium generated by photorespiration^31^. In addition to reduced levels of glutamine^13^, ammonium may also accumulate in the *acr11* mutant. Excess amounts of ammonium are toxic to plants, which may activate ROS production and trigger the downstream SA-associated defense responses (Fig. 8).

The reactions catalyzed by GS/Fd-GOGAT require ATP and reducing power derived from photosynthesis. Thus, the GS/Fd-GOGAT cycle is a strong electron sink, which plays an important role in consuming and translocating reducing equivalents inside the plant cell. Perturbation of the GS/Fd-GOGAT cycle results in Gln-deficiency and redox imbalance, which may trigger the overproduction of ROS and enhance SA-associated defense responses in the *acr11* mutant. Alternatively, ACR11 may have an effect on SA biosynthesis, and subsequently affect the accumulation of ROS. It is known that the interplay of SA and ROS can modulate the expression of defense genes^32^. Overproduction of SA may also result in increased levels of ROS and enhanced defense responses in the *acr11* mutants. It will be interesting to investigate the role of ACR11 in the interconnection of Gln metabolism, ROS production, and SA-associated defense network in Arabidopsis.

Fd-GOGAT1 has been shown to interact and regulate the activities of UDP-sulfoquinovose synthase and serine hydroxymethyltransferase^33,34^. Interestingly, Fd-GOGAT1 plays a regulatory role when interacts with these enzymes, which is independent of its catalytic function^33,34^. Thus, it is possible that the ACR11/Fd-GOGAT complex may have additional functions independent of the assimilation of photorespiratory ammonium in Arabidopsis. We cannot exclude the possibility that the accumulation of ROS and SA, and the defense-related phenotypes observed in the *acr11* mutants are not directly linked to the GS/Fd-GOGAT cycle. The ACR11 homologs are conserved from algae to land plants. Further studies on the functions of Arabidopsis ACR11 will provide insights into the molecular mechanism of ACR proteins in plants.

## Methods

### Plant materials and growth conditions

*Arabidopsis thaliana* ecotype Columbia-0 and T-DNA insertion mutants *acr11-2* (Sail_14_H10) and *acr11-3* (Salk_025722) were obtained from the Arabidopsis Biological Resource Center. Plants were grown in soil or on tissue culture plates in a controlled growth chamber on a 16-h light/8-h dark cycle at 23 °C as previously described^35^.

### Trypan blue staining and detection of ROS

Rosette leaves from 5-week-old Arabidopsis wild-type and *acr11* mutant plants were used for trypan blue, DAB, NBT, and SOSG staining as previously described with minor modifications^36,37^. The DAB (D5637, Sigma-Aldrich) staining solution, 1.25 mg/ml, was freshly prepared in sterilized water and adjusted to pH 3.8 with KOH. Detached rosette leaves were immersed and infiltrated under vacuum with DAB staining solution and then cleared in boiling 95% (v/v) ethanol for 10 min. For NBT staining, detached rosette leaves were immersed and infiltrated under vacuum with 3.5 mg/ml NBT (N6876, Sigma-Aldrich) staining solution in 10 mM potassium phosphate buffer containing 10 mM sodium azide. After vacuum infiltration, stained leaves were bleached in boiling 95% ethanol (v/v) for 10 min. The commercially available fluorescent dye SOSG (S36002, Thermo Fisher) was used to detect singlet oxygen. Rosette leaves from 5-week-old plants were infiltrated with a solution of 100 μM SOSG in 50 mM phosphate potassium buffer (pH 7.5). Plants were exposed to light for 30 min and infiltrated leaves were observed under a 510 META Zeiss confocal laser scanning microscope with excitation at 480 nm and emission at 530 nm.

### Callose staining and microscopy

Aniline blue (415049, Sigma-Aldrich) was used to stain callose deposition. Rosette leaves of 5-week-old Arabidopsis were cleared overnight in 95% ethanol (v/v) at room temperature. The completely cleared leaves were rehydrated in sterilized water, and then immersed in aniline blue staining solution of 0.01% (w/v) in 0.15 M phosphate buffer, pH 9.5, for 30 min. The callose deposition was observed under a UV illumination using Zeiss Axio Scope A1 microscope. Callose deposits were quantified by the “analyze particles” function of ImageJ (http://rsb.info.nih.gov/ij/).

### Quantitative (q) RT-PCR and RNA gel-blot analysis

Arabidopsis total RNA was isolated using a phenol extraction protocol as previously described^38^. Total RNA extracted from rosette leaves of 5-week-old Arabidopsis wild-type and *acr11* mutant plants was digested with DNase I and used for qRT-PCR analysis. All qRT-PCRs were performed with three biological repeats and the expression data were normalized to the nuclear gene *ACTIN2* (*At3g18780*). The following primers were used for qRT-PCR: *PR1* (*At2g14610*), 5′-TTCACAACCAGGCACGAGGAG-3′, 5′-GCCAGACAAGTCACCGCTACC-3′; *PR2* (*At3g57260*), 5′-CTTGAACGTCTCGCCTCCAGTC-3′, 5′-TCCAGAAACCGCGTTCTCGATG-3′; *PR5* (*At1g75040*), 5′-CAATTGCCCTACCACCGTCTGG-3′, 5′-CTTAGACCGCCACAGTCTCCG-3′; *CBP60G* (*At5g26920*), 5′-CGGGCGTAACACTTCTCTTC-3′, 5′-AGCTTCGGCCTTTAATTGGT-3′; *WRKY18* (*At4g31800*), 5′-CATACGAAGGGACGCATAAC-3′, 5′-CCTTTCGTTTTTCTCCAACG-3′; *WRKY53* (*At4g23810*), 5′-GGCAGTGTTCCAGAATCTCC-3′, 5′-GCCTCTCTCTGGGCTTATTC-3′; *ACTIN2* (*At3g18780*), 5′-GGTAACATTGTGCTCAGTGGTGG-3′, 5′-AACGACCTTAATCTTCATGCTGC-3′; *ICS1* (*At1G74710*), 5′-TGGCGAGGAGAGTGAATTTG-3′, 5′-TGGGTCACTTCCAGCTACTA-3′; *ICS2* (*At1G18870*), 5′-GTTTGCGGATGTCCAGTAGAA-3′, 5′-CCACCACCAAAGAACCCAATA-3′. For RNA gel-blot analysis, total RNA (10 μg) was separated in standard formaldehyde gel by electrophoresis and blotted onto a nylon membrane. To detect the transcripts of *ACR11* and SA marker genes, digoxigenin (DIG)-labeled single-stranded DNA probes were generated by PCR using the following primers: *ACR11* (*At1g16880*), 5′-ATGGCTATGGCCT CTGCTTC-3′, 5′-GAAACTTGACTCGTCAGTTG-3′; *PR1* (*At2g14610*), 5′- ATGAATTTTACTGGCTATTCTCG-3′, 5′-TTAGTATGGCTTCTCGTTCAC-3′; *PR2* (*At3g57260*), 5′-ATGTCTGAATCAAGGAGCTTAGC-3′, 5′-TTAGTTGAAATTAACTTCATACTTAG-3′. DIG probe labeling, pre-hybridization, hybridization, wash conditions and detection were performed according to the Boehringer-Mannheim Genius System User’s Guide: DIG Application Manual for Filter Hybridization.

### Salicylic acid (SA) measurement

Rosette leaves from 5-week-old Arabidopsis wild-type and *acr11* mutant plants were used for SA measurement. Sample extraction and quantitative analysis of free SA were performed as previously described^39^. The SA measurement was conducted by the Metabolomics Core at Academia Sinica using Ultra Performance Liquid Chromatography-High Definition Mass Spectrometry (Waters, http://www.waters.com).

### Pathogen infection assays

Five-week-old Arabidopsis wild-type and *acr11* mutant plants were used for *Pst* infection assays as previously described^40^. Arabidopsis plants were dipped in a bacterial suspension of 10^7^ colony-forming units (cfu)/mL in 10 mM MgCl2 containing 0.01% (v/v) Silwet L-77 for 15 min. For inoculation by syringe infiltration, 3–4 leaves per plant were infiltrated with bacterial suspension of 10^5^ cfu/ml using a 1-ml syringe without a needle. Plants were kept at 100% relative humidity for one day after infection, and symptoms were photographed 3 days post inoculation. For analysis of bacterial growth, 8 leaf discs with 0.5 cm diameter from 4 different plants sampled at 0 to 3 days after inoculation were used to measure bacterial growth as previously described^40^.

## Electronic supplementary material

Supplementary Information
