# Supplementary material for: ACR11 modulates levels of reactive oxygen species and salicylic acid-associated defense response in Arabidopsis
Source: Sci Rep. 2018 Aug 7;8:11851. doi: 10.1038/s41598-018-30304-0 (PMC6081435; doi:10.1038/s41598-018-30304-0)
Supplement: Supplementary file 1 — Supplementary Information [file 41598_2018_30304_MOESM1_ESM.pdf]

Supplementary Information

ACR11 modulates levels of reactive oxygen species and salicylic acid-associated defense response in Arabidopsis

Shashi Kant Singh†, Tzu-Ying Sung†, Tsui-Yun Chung†, Shao-Yu Lin, Sang-Chu Lin, Jo-Chien Liao, Wei-Yu Hsieh and Ming-Hsiun Hsieh*

Institute of Plant and Microbial Biology, Academia Sinica, Taipei 11529, Taiwan

*Corresponding author:

Ming-Hsiun Hsieh

E-mail: ming@gate.sinica.edu.tw

TEL: +886 2 2787 1168

FAX: +886 2 2782-7954

† These authors contributed equally to this work.

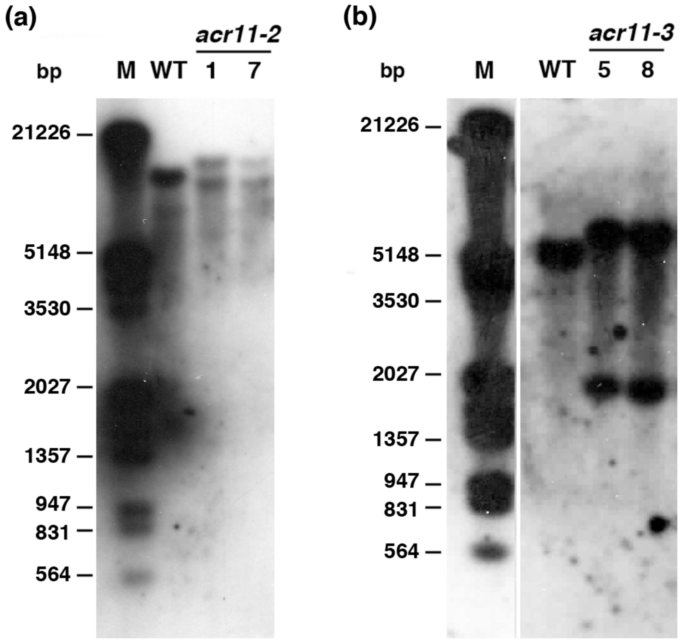

Figure S1. Southern blot analysis using digoxigenin (DIG)-labeled *ACR11* probe. (a) Genomic DNA from Arabidopsis wild-type (WT) and two *acr11-2* sibling plants (1 and 7) digested with *Bsr*GI was used for Southern blot analysis. (b) Genomic DNA from Arabidopsis WT and two *acr11-3* sibling plants (5 and 8) digested with *Mfe*I was used for Southern blot analysis. These results confirm that the *acr11-2* and *acr11-3* mutants are homozygous. The primers used to generate the DIG-labeled *ACR11* probe are described in Methods. M, DIG-labeled molecular weight marker.

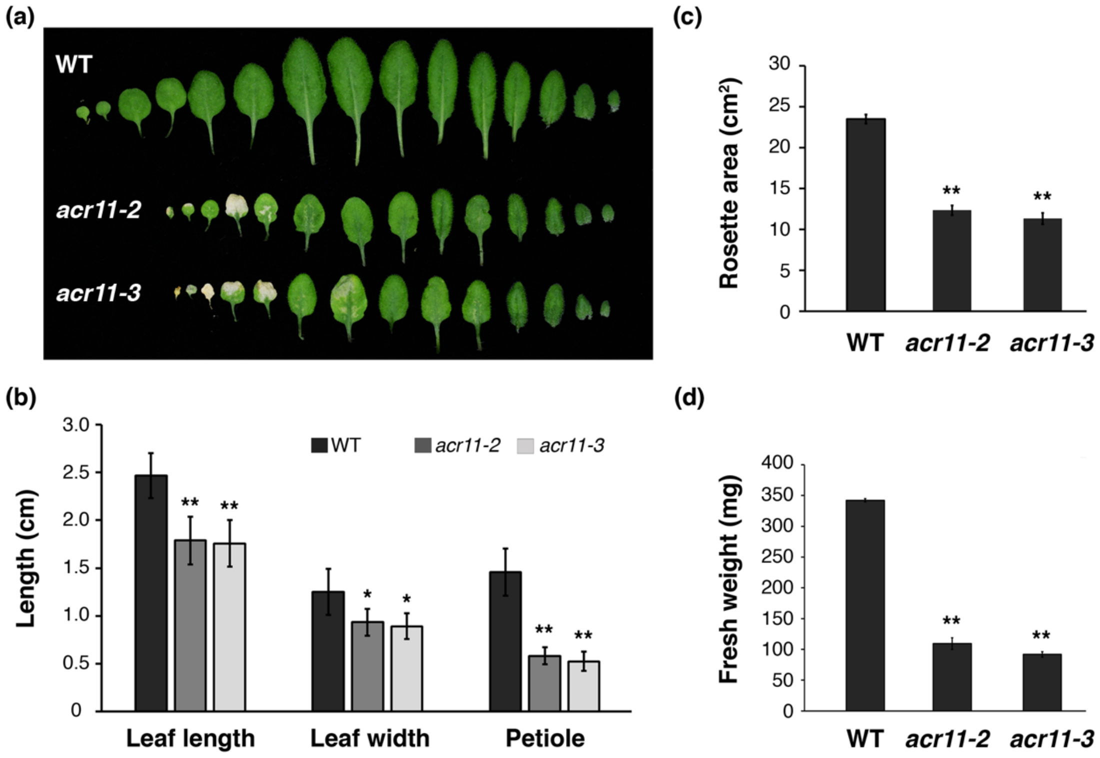

Figure S2. Phenotypic analysis of Arabidopsis *acr11* mutants. (a) Leaves from 5-week-old Arabidopsis wild-type (WT) and *acr11* mutant plants. (b-d) Comparison of leaf length (b), rosette area (c), and fresh weight (d) between 5-week-old Arabidopsis wild-type (WT) and *acr11* mutant plants. Asterisks indicate significant differences (*$P < 0.05$; **$P < 0.01$; Student's *t* test) compared to the WT.

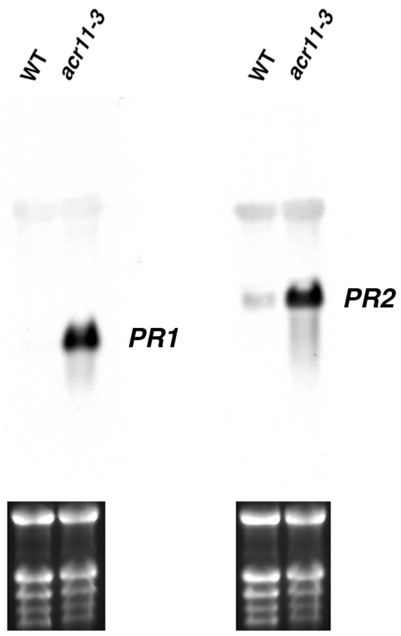

Figure S3. RNA gel-blot analysis. Total RNA extracted from rosette leaves of 5-week-old Arabidopsis wild-type (WT) and *acr11-3* mutant plants was used for RNA gel blot analysis to detect the expression of *PR1* and *PR2*.

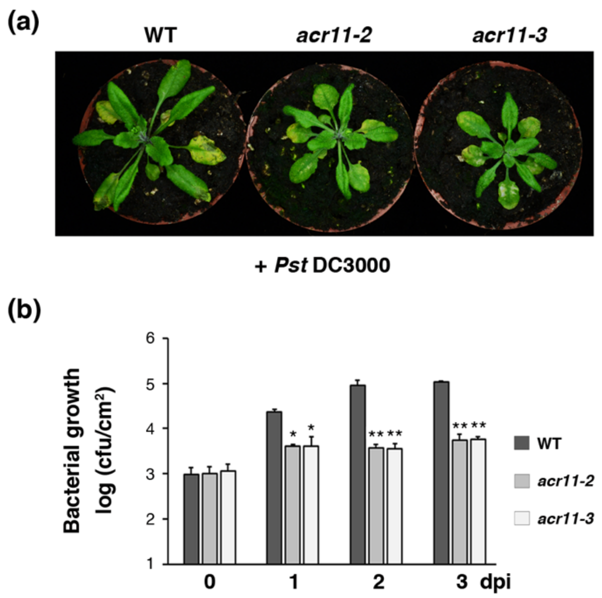

Figure S4. Enhanced disease resistance in the *acr11* mutants. (a) Symptoms of wild-type (WT), *acr11-2* and *acr11-3* rosette leaves 3 days after inoculation with *Pseudomonas syringae* pv. *tomato* DC3000 (*Pst*) by dipping. (b) Growth of *Pst* in Arabidopsis WT, *acr11-2* and *acr11-3* mutants. Bacterial titers were evaluated at 0 to 3 days post inoculation (dpi). Results are means ± SD from three independent experiments. Asterisks indicate significant differences (*$P < 0.05$; **$P < 0.01$; Student's t test) compared to the WT.

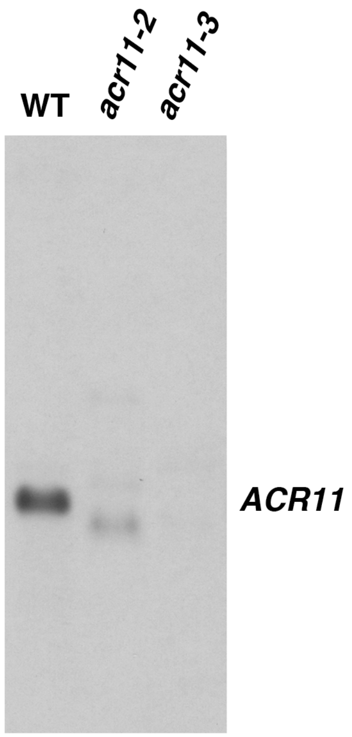

Figure S5. RNA gel-blot analysis to detect the transcripts of *ACR11* in Arabidopsis wild-type (WT), *acr11-2*, and *acr11-3* seedlings.
